# Changes in human intervertebral disc biochemical composition and bony end plates between middle and old age

**DOI:** 10.1371/journal.pone.0203932

**Published:** 2018-09-18

**Authors:** Delio Eulalio Martins, Valquiria Pereira de Medeiros, Marcelo Wajchenberg, Edgar Julian Paredes-Gamero, Marcelo Lima, Rejane Daniele Reginato, Helena Bonciani Nader, Eduardo Barros Puertas, Flavio Faloppa

**Affiliations:** 1 Department of Orthopaedics and Traumatology, Universidade Federal de Sao Paulo–UNIFESP, Sao Paulo, SP, Brazil; 2 Department of Biochemistry, Universidade Federal de Juiz de Fora, Juiz de Fora, MG, Brazil; 3 Faculty of Pharmaceutical Science, Universidade Federal de Mato Grosso do Sul, Campo Grande, MS, Brazil; 4 Department of Biochemistry, Universidade Federal de Sao Paulo–UNIFESP, Sao Paulo, SP, Brazil; 5 Department of Morphology and Genetics, Universidade Federal de Sao Paulo–UNIFESP, Sao Paulo, SP, Brazil; University of Pennsylvania, UNITED STATES

## Abstract

**Objective:**

This study evaluates molecular, nutritional and biochemical alterations in human intervertebral discs between middle and old age.

**Methods:**

Twenty-eight human lumbar intervertebral discs from donors were evaluated and separated into two groups: Middle-aged (35–50 years old, relatively non-degenerate discs of Pfirrmann grades 1–3, n = 15) and Old-aged (≥80 years old, all degenerate Pfirrmann grade 4 or 5, n = 13). Parameters which might be expected to to be related to nutrient supply and so the health of disc cells (eg the porosity of the vertebral endplate, cell viability and cell density) and to disc extracellular composition (ie quantification of glycosaminoglycan disaccharides and hyaluronic acid molecular weight) and collagen organization, were analyzed. Three regions of the intervertebral disc (anterior annulus fibrosus, nucleus pulposus, and posterior annulus fibrosus) were examined.

**Results:**

The old-aged group showed a decrease in content of sulphated and non-sulphated glycosaminoglycans relative to middle-aged and there were also alterations in the proportion of GAG disaccharides and a decrease of collagen fiber size. Hyaluronic acid molecular weight was around 200 kDa in all regions and ages studied. The anterior annulus differed from the posterior annulus particularly in relation to cell density and GAG content. Additionally, there were changes in the bony endplate, with fewer openings observed in the caudal than cranial endplates of all discs in both groups.

**Conclusions:**

Results show the cranial vertebral endplate is the main vascular source for the intervertebral discs. Hylauronic acid molecular weight is the same through the intervertebral disc after age of 50 years.

## Introduction

**Fig 1:**
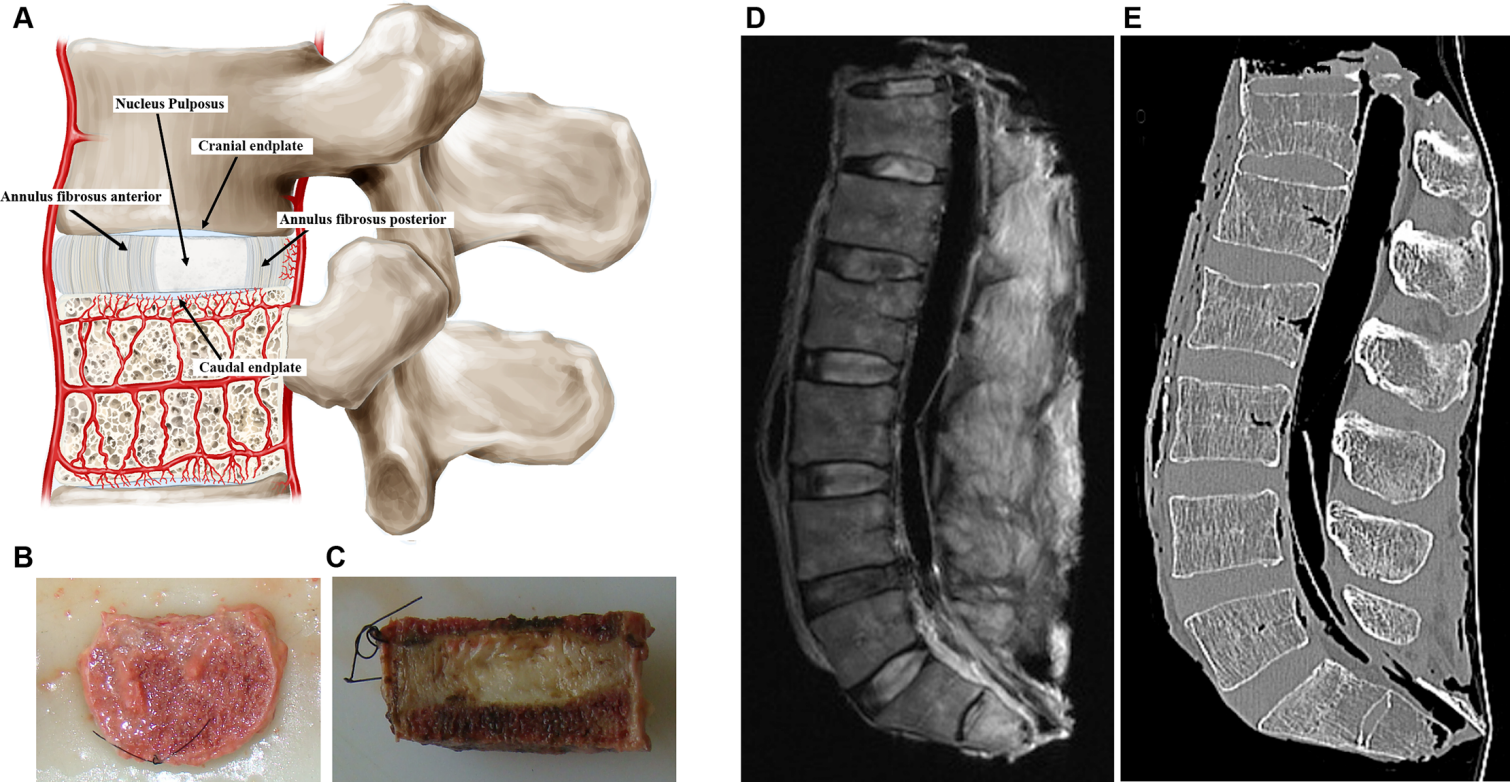
(A) schematic drawing representative of functional spine unit and regions of the disc showing the caudal and cranial cartilaginous endplates, the nucleus pulposus and the anterior and posterior annulus fibrosus. The figure also shows the blood supply to the disc (B) Superior view of a spine unit after the removal of the posterior arch. (C) Sagittal view of the mid part of the spine unit, note the nylon stitch in the antero-superior region to maintain the fragment in functional position in all steps of the study. (D) MRI and (E) CT sagittal view with ligaments and paraspinal muscles intact for better contrast.

The intervertebral discs are the biggest avascular structure in the human body. They lie between the vertebral bodies and make up around one third of the height of spinal column. The discs consist of two regions with the central, more gelatinous nucleus pulposus (NP), surrounded by a fibrous ring, the annulus fibrosus (AF). The discs are separated from the adjacent vertebrae by a thin layer of hyaline cartilaginous tissue, the cartilage endplates (Fig 1).

The composition of the different regions of the disc differ, with the NP consisting mainly of a high concentration of proteoglycans and water, embedded in a loose collagen network, while the AF has a high concentration of fibrillar collagens, organised into concentric lamellae. The disc also has a small population of resident cells which make and maintain proteoglycans, collagens and other matrix components[1,2]. The organisation and composition of the extracellular matrix of the intervertebral disc enables it to fulfil its main roles of providing flexibility to the spinal column and carrying the high loads arising during daily activities.

Degeneration of the disc, resulting in degradation of matrix macromolecules and loss of structural integrity, has been considered a major cause of low back pain[3]. Proteoglycan loss is one of the early features of disc degeneration[2] and are key to the loadbearing properties of the disc as they regulate disc hydration[4]; several aspects of the proteoglycan biochemistry such as their constituent chondroitin sulphate chains, that determine disc osmolarity[5] and hyaluronan content, which affects proteoglycan aggregation[4] are important to the disc function. Lumbar back pain has an overall prevalence of 60–80% varying in different age groups and the older population is the group with more frequent episodes of low back pain[6]. However, much remains unknown about its aetiology. Twin studies have found that genotype has a much stronger influence than abnormal mechanical loading or smoking on the development of degeneration[7]. A factor which may determine progression of degeneration, is a fall in disc nutrient supply[8–10] that may promote the loss of cell viability and hence failure to synthesize the disc’s macromolecules. The viability of disc cells depends on the supply of nutrients, which are mainly provided by blood vessels at the disc-endplate interface, where they penetrate the subchondral space through marrow spaces seen as openings in the bony endplate[11]. Nutrients diffuse from these blood vessels via openings in the subchondral plate, through the cartilaginous endplate and the extracellular matrix of this and the disc to the cells. This peculiarity of the disc vasculature results in low concentration of cells, in comparison to other tissues, especially in the NP[2]. With ageing and degeneration, the transport pathway at the endplate is compromised so that nutrient supply and hence cell viability and extra cellular matrix turnover are adversely affected.

Ageing is the highest risk factor for disc degeneration[12] that seems to start in the second decade with clefts in the NP[13]. Collagen and proteoglycans also decrease during aging[14] however, while structural and biochemical changes with degeneration and ageing have been studied in the nucleus pulposus (NP)[4], less is known about changes in the annulus fibrosus (AF).

Changes over ageing have been relatively ignored, even though over 40% of people above 70 years of age have highly degenerate discs[12] and disc degeneration predisposes sufferers to development of clinical syndromes which particularly affect the elderly[15] such as spinal stenosis and hyperkyphosis. There are reported asymmetries between the cranial and caudal endplates in regard to trabecular architecture[16], thickness and bone mineral density[17] which vary according to aging[17,18], but how the endplates alter in the elderly and relate to viability of disc cells has not been systematically investigated.

How these properties alter in the older population is a gap in our knowledge base, despite it being likely to be important for understanding the aetiopathogenesis of disc degeneration with its consequent impact on clinical disorders such as spinal stenosis, the development of which are so prominent between middle age and old age. Thus, the objective of this study was to evaluate molecular, nutritional and biochemical alterations in human intervertebral discs between middle and old age focusing particularly on changes in proteoglycan and bony endplate.

## Materials and methods

Institutional Review Board of Universidade Federal de Sao Paulo (0151/09) has approved the study before starting. Samples were obtained from cadavers during routine necropsy to verify the cause of death at the City’s Death Checking Service after making personal contact with a family member and had the informed consent signed.

All reagents were obtained from ‘Sigma Chemical Company, St Louis, USA’ unless otherwise stated.

### Collection of spinal columns from donors

A cadaveric study was carried out with 28 lumbar intervertebral discs that were harvested from ten donor human cadavers within 24 h of death and without any known spinal diseases (fracture, infection, tumour, spinal deformities, previous spinal surgery or metabolic diseases) (Table 1). Spines were separated into two groups with middle-aged group, from individuals less than 50-years of age (mean 43.6±6.02, range 34–49) and old-aged group, 80-years of age or more (mean 85±4.7, range 80–91).

**Table 1:** Details of donor samples in the two groups studied.

Patient	Gender	Race	Age	Nº of Discs	Cause of Death	Smoking	Alcohol	BMI
**Middle-aged**								
1	M	W	49	4	Bronchopneumonia	Yes	No	17.3
2	M	W	45	3	Bronchopneumonia	No	No	18.6
3	M	W	48	3	Myocardial Infarction	No	No	27.0
4	F	W	34	2	Peritonitis	Yes	Yes	23.7
5	F	W	42	3	Aortic aneurysm	Yes	Yes	26.9
Mean			43.6					22.7
SD			6.02					4.56
**Old-aged**								
6	M	W	82	3	Bronchopneumonia	No	No	20.2
7	F	W	89	3	Myocardial Infarction	No	No	17.5
8	F	W	91	3	Myocardial Infarction	No	No	21.1
9	M	W	83	2	Myocardial Infarction	No	No	31.5
10	M	W	80	2	Bronchopneumonia	Yes	Yes	22.8
Mean			91					22.6
SD			4.74					5.32

(M) male; (F) female; (W) white. Age (years); (BMI) body mass index (kg/m^2^); (SD) standard-deviation

Spine samples were removed through an anterior approach and wrapped in a plastic sealed bag and inserted in a second bag with a saline-soaked gauze to prevent dehydration, prior to being transported inside an ice-cooled box to the imaging facility where a CT scan and MRI were accomplished. Ligaments and paraspinal muscles were left intact for better contrast during images and to minimize dehydration. The intervertebral discs samples were kept at -4°C for 4–12 hours depending on the time of the day that they were collected from donors, until dissection. The specimens were then separated in functional spine units consisting of two vertebrae surrounding one disc (Fig 1).

### Preparation of intervertebral discs

A 5 mm mid-center coronal section was cut from the spinal units listed (Table 1) using a band-saw. This included the anterior annulus fibrosus (AFa), NP, and posterior annulus fibrosus (AFp) of the disc, enclosed between a portion of the superior and inferior vertebral bodies (Fig 1C). Two further 5 mm thick sections adjacent to this mid central section were cut in a similar way for histological and biochemical purposes, respectively. For biochemical analysis, the discs were separated into three regions: AFa, NP and AFp. A nylon stitch was placed in the antero-superior region of each fragment to clearly mark the location in all steps of the study.

### Imaging

MR images of the spinal columns were obtained using a spine surface coil on a Siemens AG2006 1.5 Tesla Magnetic Resonance Imager (Syngo version MRA30^®^, model Sonata Maestro-Class, software NUMARIS/4). Sagittal and axial views were obtained using T1-weighted and T2-weighted sequences with 4 mm slice thickness and 0.6 and 0.4 mm gaps for sagittal and axial sequences, respectively. Field-of-view for the sagittal sequence was 270 and 200 for the axial slices.

CT scans of the spinal columns were accomplished using a Brilliance 64-slice-CT-scanner (Philips, Cleveland, USA) and images were obtained at 1 mm thickness and 1 mm gap. Three observers in consensus (two orthopedic surgeons and one musculoskeletal radiologist) analysed at the same time all images to exclude any pathology, and classified discs according to the Pfirrmann classification[19] (Table 2) where Grade 1 discs are young and healthy, and Grade 5 discs are severely degenerate.

**Table 2:** Intervertebral disc degenerative status classified by the Pfirrmann scale.

Middle-aged			Old-aged		
Donor	Disc Level	Pfirrmann	Donor	Disc Level	Pfirrmann
1	L1-L2	IV*	6	L1-L2	IV
	L2-L3	II		L2-L3	III*
	L3-L4	II		L3-L4	IV
	L4-L5	III		L4-L5	V
	L5-S1	II		L5-S1	IV*
2	L3-L4	II	7	L3-L4	IV
	L4-L5	II		L4-L5	IV
	L5-S1	I		L5-S1	V
3	L3-L4	I	8	L3-L4	IV
	L4-L5	II		L4-L5	V
	L5-S1	II		L5-S1	IV
4	L3-L4	III	9	L3-L4	IV
	L4-L5	IV*		L4-L5	IV
	L5-S1	II		L5-S1	III*
5	L3-L4	II	10	L3-L4	II*
	L4-L5	III		L4-L5	IV
	L5-S1	III		L5-S1	IV

(*) levels which were not included in the analysis

### Cell viability

Cell viability in the 5 mm disc sections was measured using a tetrazolium assay[20] (S1 and S2 Figs). The disc from the central section of each functional spinal unit was dissected from the bone using a scalpel. The disc sections were incubated for 18 h in 3-(4,5-dimethylthiazol-2-yl)-2,5-diphenyl-tetrazolium bromide (MTT) (0.5 mg/ml at 37°C) in low-glucose Dulbecco’s Modified Eagle’s Medium (catalog 31600–034, Gibco, Gran Island, NY, USA), 100 U/ml penicillin, and 100 μg/ml streptomycin (catalog 1414122, Gibco, Grand Island, NY, USA). Following incubation, the disc was dissected into AFa, NP and AFp. The three discs regions were snap-frozen and 20 μm thick sections were cut using a cryo-microtome (HM550 Microm^®^/Carl Zeiss) onto slides and mounted with Vectashield^®^ medium with DAPI (Vector Laboratories Inc., Peterborough, UK) that stains DNA and hence can be used to obtain total number of cells, both live and dead. Representative images were acquired using a confocal microscope with multiphoton titanium-sapphire laser (LSM780^®^/Carl Zeiss) with excitation at 720 mm and emission collected at 420–470 mm and light transmission image (S1 Fig). Two observers each counted at least 200 cells per section and calculated the percent of cells which stained positively for the formazan product of the tetrazolium MTT (indicating viable cells).

### Endplate porosity and microarchitecture

**Fig 2:**
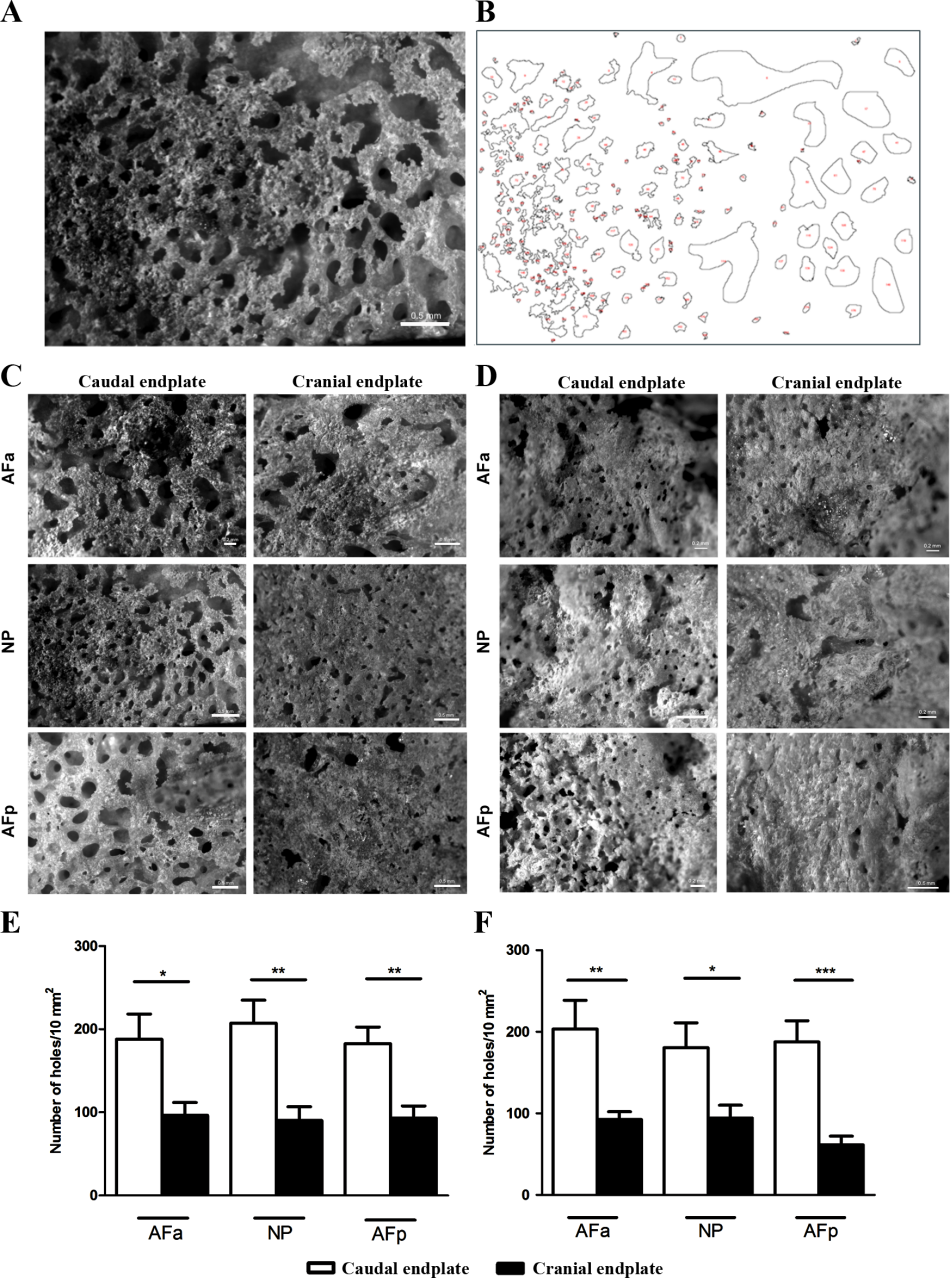
Endplate openings in the different regions of the disc per group. In (A) an example of a scanning image from endplate openings and in (B) an example of a binary image of a nucleus pulposus region in the ImageJ software. (C) Middle-aged (younger than 50-years of age) and (D) Old-aged (older than 80-years of age). Observe that in caudal endplates there are more openings in all regions. Endplate openings per 10 mm^2^ in cranial and caudal endplates above and below the discs of Middle-aged (E) and Old-aged (F). Images acquired from a 10 mm^2^ area in the Stereo Microscope Discovery V.8. AFa–anterior annulus fibrosus; NP–nucleus pulposus; AFp–posterior annulus fibrosus. (*) p < 0.05; (**) p < 0.01; (***) p < 0.001. ANOVA-test, followed by Bonferroni post-test.

Samples of 5 mm thick of the central region of the vertebra, encompassing the entire extension from anterior to posterior were enzymatically treated with 10 mg/ml papain (USB Corporation, Cleveland, Ohio, USA) at 65°C for seven days to remove adhering extracellular matrix, following a modification of the Benneker protocol[9]. The endplates were then cleaned with a soft pulsatile water jet, degreased in 1% Triton X-100 and dried at 37°C for 24 h. Images of 10 mm^2^ area were acquired using a Stereo Microscope Discovery^®^ V.8 (Carl Zeiss) with extended focus and a 1.4 megapixels Axiocam with objective Plan-Apochromat S 1.0x. Images were collected in the center of the NP area, at a midpoint between the NP and both the AFa and AFp and also at a distance of 5 mm from the edge. The images were converted to gray-scale mode. A binary image at a fixed intensity level was created and analysed using the ImageJ^®^ software (US National Institutes of Health, Bethesda, Maryland, USA, http://imagej.nih.gov/ij) (Fig 2A and 2B). Openings smaller than 5 μm^2^ and bigger than 100 μm^2^ were excluded from analysis to avoid openings that could be due to reflections or to irregularities in endplate surface such as Schmorl nodes, fracture lesions or erosion[18].

### Biochemical analysis

For biochemical analysis, one of the 5 mm thick adjacent sections from each disc examined was separated into three regions (AFa, NP and AFp) according to the macroscopic difference in lamellar structure characteristic of these regions. Samples were dried to constant weight at 100°C.

All tissue samples were digested overnight in 5 ml 1% papain (USB Corporation, Cleveland, Ohio, USA) in sodium-free buffer[21] at 65°C. The same set of samples was used to measure the amount of sulphated glycosaminoglycans (S-GAG), characterize chondroitin sulphate (CS) disaccharides, quantitate hyaluronic acid (HA) and analyse its molecular weight (MW). An aliquot of 1 ml of the supernatant was used for analysis of GAGs by its precipitation with five volumes of methanol (-20°C, overnight)[22,23]. Five micrograms of the precipitate and a 5μl aqueous mixture of 1 mg/ml of standard S-GAGs (chondroitin-4-sulphate from whale cartilage, chondroitin-6-sulphate from shark cartilage, dermatan sulphate (DS) from bovine intestinal mucosa (Seikagaku Kogyo, Tokyo, Japan), and heparan sulphate from bovine lung (extracted and purified by the Molecular Biology Division, Federal University of Sao Paulo, Brazil)) were analysed by electrophoresis following the protocol of Dietrich and Dietrich[22]. The electrophoretic band intensities were quantified by densitometry at 525 nm with a 5% error margin compared to a known content of the standard S-GAG. The identities of S-GAG were characterized by treatment of the GAG precipitate with chondroitin ABC lyase from *Proteus vulgaris* (Seikagaku America) and chondroitinase AC enzyme (S3 Fig).

For disaccharide analysis, 200 μl of a pool of five discs was treated by CS ABC lyase was desalted using PD MidiTrap G-10 (GE Healthcare Bio-Sciences AB, Uppsala, Sweden) gravity mini-columns. The disaccharide identification was done on a 150x4.6 mm Phenosphere SAX column (Phenomenex, Torrance, CA, USA), using a NaCl gradient of 0.1M during 30 min with 1 ml/min flux and UV detection at 232 nm, at room temperature[24]. The chromatograms were compared to the elution profile of CS/DS disaccharide standards. Results were expressed as percentages.

HA was quantified in a separate 1 ml sample of the papain digest using a highly specific fluorimetric enzyme-linked immunosorbent assay (ELISA)[25]. Furthermore, hyaluronic acid molecular weight (HA-MW) measurement was obtained in a separate 100 μl sample, via high-pressure liquid chromatography on a 300x8 mm Shodex OHpak SB-805HQ (Phenomenex) coupled to a 300x8 mm Shodex Ohpak SB-804HQ (Phenomenex), at room temperature. The mobile phase was 0.2M NaCl. The flow rate was kept at 0.5 ml/min over 60 min with UV detection at 205 nm. Fractions of 0.2 ml were collected for HA quantitation as previously described[25]. The column was previously calibrated with monodisperse HA standards (Hyolase, USA) of known MWs: 2500 kDa, 601 kDa, 250 kDa, 150 kDa and 100 kDa (S4 Fig).

### Histological analysis

The final 5 mm thick coronal section was submitted to a routine histopathologic procedure: fixed in buffered 4% formaldehyde, decalcified for 90 days in 25% formic acid, pH 2.0; dehydrated and paraffin embedded and 5 μm-thick sections were prepared. Sections were immersed in a 0.2% phosphomolybdic acid solution, washed in distilled water, and incubated in 0.1% Sirius Red diluted in saturated picric acid solution for 1 h at room temperature according to a previous protocol allowing the visualization of collagen network by birefringence intensity[23]. Picrosirius red is a histochemical technique to analyze the distribution and quantitative estimation of collagen fibers.[26]

The sections were examined by polarization microscopy with an AxioScope A1 microscope (Carl Zeiss). Images were captured with a 5x objective lens against a black background and evaluated with ImageJ^®^ after split imaging in channels (red–green–blue). Images were analyzed using the “measure” option (HIH public domain software; http://rsbweb.nih.gov/ij/). to evaluate the relationship between reddish/greenish fibers[23].

### Statistical analysis

Variables were tested for normality using the Kolmogorov-Smirnov test. Results were expressed as mean±standard error. Differences were evaluated using parametric analyses. The chi-square test or Fisher’s exact test were used to analyse frequency distributions. All statistical tests were considered statistically significant at the level of 5%. Data analysis was performed using SPSS software (SPSS, Version 17.0, SPSS, Chicago, USA).

## Results

Disc donors were divided into two groups by age: middle-aged (35–50 years) and old-aged (over 80 years). All donors were Caucasians and details of the respective disc levels analysed, cause of death, BMI, alcohol and smoking habits and their Pfirrmann[19] classification are given in Tables 1 and 2. Body mass index did not vary between groups (Middle-aged = 22.7 Kg/m2; Old-aged = 22.6 Kg/m2). Of the 28 discs, according to Pfirrmann, two were of disc degeneration grade 1, nine of grade 2, four of grade 3; ten of grade 4 and three of grade 5. Middle-aged contained 15 discs of grades I-III and old-aged contained 13 discs which were all grade IV or V discs.

### Endplate openings

Fig 2C and 2D show images of caudal and cranial bony endplates for both groups after enzymatic removal of the surrounding tissue. Quantification of endplate openings found that the caudal endplate had significantly more openings than the samples from the cranial endplate for the all three regions and for both groups (Fig 2E and 2F). However, apart from for AFp (p<0.05), there was no significant difference between the two age groups in the same region.

### Cellularity and cell viability

Images of viable and total cells in both groups are available in supporting information files (S2 Fig). Cell counts of these images, found that the total number of cells/mm^2^ was greater in the NP and AFp regions of middle-aged than old-aged discs (Table 3). In both disc groups, the highest number of cells/area was seen in the AFp region. A significant proportion of the cells were dead, with only 40–65% viable. Percentage of viability was greater in old-aged than in middle-aged discs in the NP and AFp though not reaching levels of significance between groups (Middle-aged: AFa 45.06±14; NP 47.09±16; AFp 41.09±14/Old-aged 2: AFa 47.37±9; NP 59.53±4,4; AFp 65.85±7; p>0.05). However, the number of viable cells/area was greater in the NP of the middle-aged discs and this is important because the number of viable cells is the critical factor in relation to disc health.

**Table 3:** Total number of cells/mm^2^, % viable cells, and total number of viable cells/mm^2^ in each region of the disc for Middle- and Old-aged discs.

	AFa			NP			AFp		
	Total cells/mm^2^	%Viable cells	Total viable cells/mm^2^	Total cells/mm^2^	%Viable cells	Total viable cells/mm^2^	Total cells/mm^2^	%Viable cells	Total viable cells/mm^2^
Middle-aged	188.4±30	45.06±14	84.78	177±12	47.09±16	83.19	499±164	41.09±14	204.59
Old-aged	188.8±18	47.37±9	89.4912	115±13	59.53±4,4	67.85	362±35	65.85±7	240.24

(AFa) anterior annulus fibrosus, (NP) nucleus pulposus, (AFp) posterior annulus fibrosus

### Biochemical analysis

#### Sulphated glycosaminoglycans and hyaluronan

**Fig 3:**
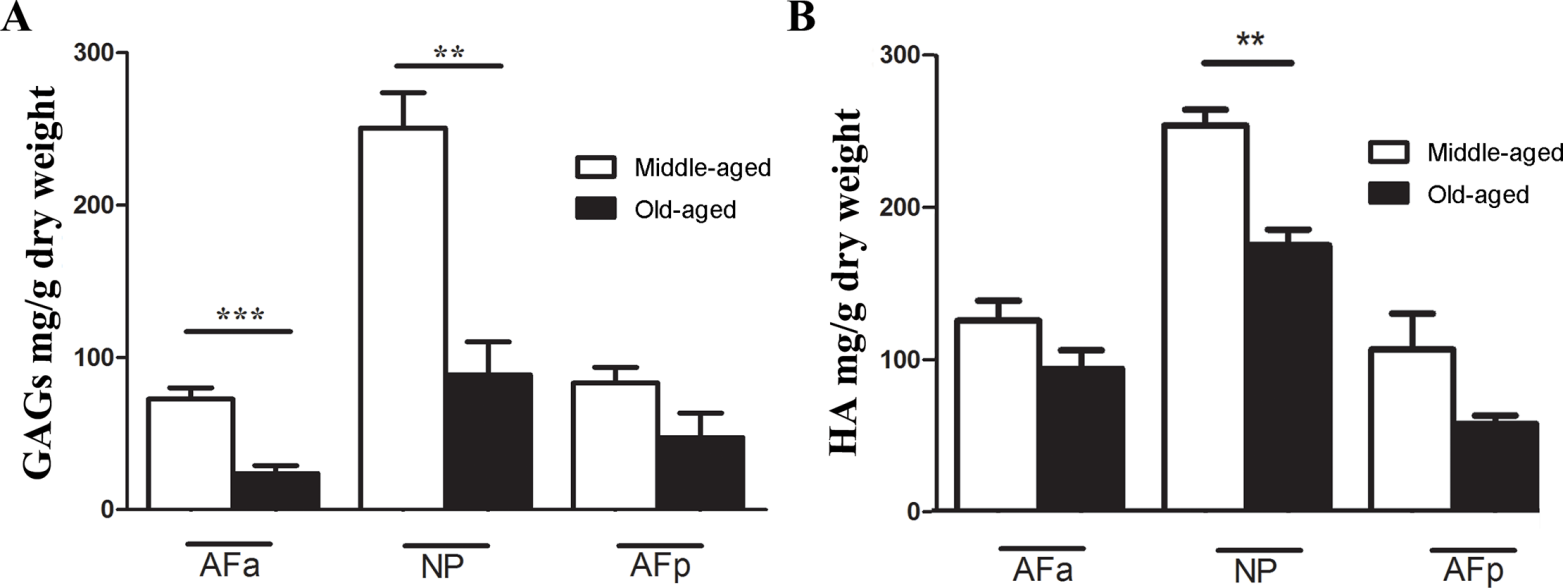
(A) Sulphated glycosaminoglycans in different regions of the disc for both groups. Observe the lower contents for old-aged group compared to middle-aged in all regions. In (B) observe the similar distribution of hyaluronic acid. (*) p < 0.05; (**) p < 0.01. ANOVA-test, followed by Bonferroni post-test.

The concentration of S-GAG and of HA was greater in the NP than AF for both groups (Fig 3). S-GAG was significantly lower in old-aged group for AFa and NP regions (Fig 3A). HA concentration of old-aged samples was only significantly lower than that of middle-aged samples in the NP region (p<0.01) (Fig 3B). This led us to investigate whether further changes could be detected in the molecular weight distribution of HA. The results showed the same molecular weight profile for all regions and groups with a mean MW of 200 kDa (S5 Fig).

The degree of sulphation of chondroitin sulphate was investigated by the analysis of its disaccharides. Changes were rather variable between groups and regions but higher levels of 6-O-sulphation are observed in the aged group for both AFa and AFp regions. As for the AFp region, sulphation levels at both 4 and 6-position decreased in old-aged and, as expected, an increase in the Di-0S (2-acetamido-2deoxy-3-O-(β-D-gluco-4-enepyranosyluronicacid)-D-galactose) (Table 4).

**Table 4:** Percentage of disaccharides products formed by the action of chondroitinase AC on glycosaminoglycan of human lumbar intervertebral disc.

	AFa		NP		AFp	
	M-A	O-A	M-A	O-A	M-A	O-A
**Di-6S**	36.14	41.24	37.28	45.37	39.28	38.43
**Di-4S**	31.94	27.74	26.58	30.77	28.78	26.48
**Di-0S**	29.96	31.02	36.14	23.86	31.94	35.09

(AFa) anterior annulus fibrosus; (NP) nucleus pulposus; (AFp) posterior annulus fibrosus. (M-A) Middle-aged; (O-A) Old-aged; (Di-0S) 2-acetamido-2deoxy-3-O-(β-D-gluco-4-enepyranosyluronicacid)-D-galactose; (Di-4S) 2-acetamido-2deoxy-3-O-(β-D-gluco-4-enepyranosyluronicacid)-4-O-sulfo-D-galactose; (Di-6S) 2-acetamido-2deoxy-3-O-(β-D-gluco-4-enepyranosyluronic acid)-6-O-sulfo-D-galactose.

#### Collagen birefringence

It is known that immature and mature collagen fibrils are differentiated by their colours under polarized light[27]. Against a black background, thick fibres are mainly type I mature collagen, consequently present intense birefringence of yellow to red colour, while thin fibrils formed mainly by type I immature collagen (including procollagen, intermediaries, and even altered collagen) and display a weak birefringence of greenish colour[27–29]. Collagen birefringence changes also can be attributed to orientation of the fibres[30]. In addition, greenish birefringence is also associated with accumulation of type III collagen[31].

**Fig 4:**
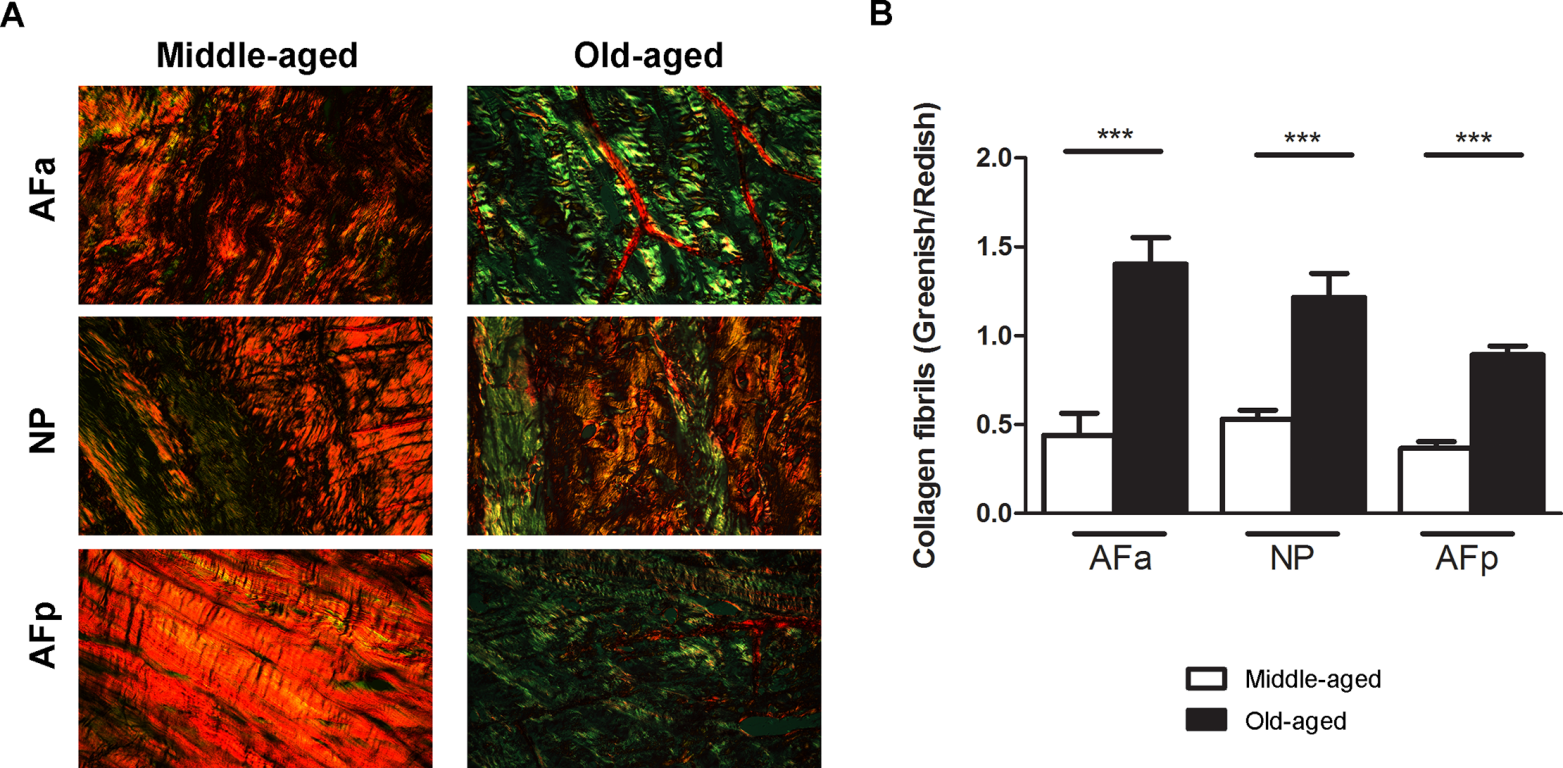
(A) Histological analysis of the human lumbar intervertebral disc stained using picrosirius red and visualized by polarized light microscopy. Observe that in all regions of the disc, in middle-aged, there is a predominance of thick fibres or reddish fibres. In (B), shows the ratio of reddish/greenish collagen fibres identified by split imaging in channels (red-green) and quantified using Image J software. AFa–anterior annulus fibrosus; NP–nucleus pulposus; AFp–posterior annulus fibrosus. (***) p < 0.001. ANOVA-test, followed by Bonferroni post-test.

Birefringence intensity evaluation of collagen fibrils under polarized light shows the predominance of orange to reddish-orange fibres in the middle-aged discs, representing thick fibres (1.6–2.4 μm; particularly in the AF), whereas, in old-aged, the majority were greenish or yellowish-green, characteristic of thin fibres (0.8 μm or less)[23,32] (Fig 4). The birefringence ratio of greenish/reddish collagen fibrils in the two groups demonstrate a significant difference, being thinner in older, more degenerate discs in all regions of the disc (p<0.001) (Fig 4).

## Discussion

We analysed separately the NP, AFa and AFp, and caudal and cranial endplates in middle-aged and old-aged intervertebral discs. We found significant differences between middle and old age in degeneration grade and in concentrations of sulphated proteoglycans and hyaluronan (Fig 3) which were both, in general, higher in middle age (Group 1) than in the old age (Group 2) discs which were severely degenerate. The cell density was greater in middle-aged discs than in old-aged discs; a large proportion of cells were non-viable (40–60%) in all discs, with viability greater in old-aged discs however the number of viable cells/area was greater in the NP of the middle-aged discs suggesting that the number of viable cells is the critical factor in relation to disc health. There were differences between posterior and anterior annulus tissue, with the posterior annulus having a notably higher concentration of cells that the anterior annulus, whereas the concentrations of S-GAGs was higher in this region (Fig 3). For both groups and all regions, the caudal bony endplates had significantly more marrow contacts with the disc than the cranial endplates (Fig 2), but there was little change in porosity between middle and old age. These results thus confirm that when analysing the disc and surrounding tissues, both age/degeneration and disc region need to be taken into account.

Changes in the vertebral marrow contacts (Fig 2) appear important for disc health as they influence transport of nutrients into the disc[11]. We however saw no significant effect of age (and degree of degeneration) on the number of marrow contacts/endplate area. Thus our results are not in agreement with those of Benneker[9] who found a strong effect of degeneration on marrow contacts, although he measured density of openings rather than number. MRI studies have examined diffusion of contrast agent into the disc and show that this varies with degree of disc degeneration[33], differs between caudal and cranial endplates, and is regulated by porosity of the endplate[34]. The higher number of marrow contacts in the caudal than in the cranial endplate could explain the results of the transport studies of Arpinar et al[34] who found a consistently higher finding in the cranial than in the caudal endplate of degenerated discs. However, there has been no report which has compared the number of marrow contacts in the cranial endplate with that of the caudal endplate of the same discs. Here, we demonstrated that the caudal endplate has more marrow contacts with the disc in both middle-age and old-age donors than the cranial endplate. Caudal endplates have lower bone mineral density and are thinner than the cranial endplate[17] and the presence of more holes in this area could contribute to the small thickness of this endplate and its susceptibility to fractures[16].

The disc of the same region (lumbar) and same specimen used to analyse the endplate openings was employed to analyse the cellular viability and number. There is little data on cell number and none on viability of human disc cells, however it should be noted that separating the slabs of the functional unit using a band bone saw could have damaged some cells and led to loss viability. Viability percentage was greater in old-aged discs but did not reach levels of significance while the number of viable cells/area was greater in the NP of the middle-aged discs. The total cell density was very low in both groups, particularly the older group, and was lowest in the NP, in agreement with previously studies[8,35]. Unfortunately, as each of the two different groups contained mainly discs of similar degeneration grades, it was not possible to relate cell viability to degeneration in this study.

We observed that not only the amount of GAG but also the substitution pattern of chondroitin changed with location and ageing (Fig 3, Table 4). Previous reports demonstrate that Di-6S is the most abundant disaccharide in children[36] but in the adult intervertebral disc the proportion of Di-4S increases[37]. Our results found that Di-6S/Di-4S increased with age (Table 4) in agreement with the results of Olczyk[37]. However, here we observed that in the NP, Di-6S was present in higher concentration only in the old-age group. Chondroitin-6-sulphate seems to be related to the maintenance of articular surfaces[38] and understanding the distribution of Di-4S and Di-6S in various human cartilages may also help in the knowledge of the pathogenesis of some heritable disorders involving the sulphation of chondroitin[39]. A significant proportion of GAGs present unsulphated regions and as sulphation of GAGs provides the osmotic pressure, necessary for maintaining disc hydration, fall in its degree of sulphation would affect disc biomechanical function. Moreover, as DMMB (1,9-dimethylmethylene blue) assay commonly used for GAGs measurement is not selective for GAGs, so the overall changes in GAGs with age is not commonly assessed by this assay in many of other studies.

The fall in the amount of HA could result from a reduction in its MW. In human articular cartilage the HA-MW is around 6000 kDa; some authors suggest that high HA-MW has a chondroprotective effect in osteoarthritic cartilage[40,41] as in the arthritic knee it plunges to 500–3000 kDa[40]. The importance of low concentration of HA during aging and its association with a lower amount of HA in cartilage degeneration suggest that this relationship may be an important factor in the age-related deterioration of knee articular cartilage[42]. We found that the HA-MW in all regions of the disc and in all ages, was around 200 kDa. However, other authors suggest that NP and AF cell numbers in culture in vitro were highest upon polyethylene glycol hydrogels formed from lower- HA-MW[43]. Thus, possibly responses of intervertebral disc cells differ from those of articular chondrocytes, and the low MW of HA within the disc helps the maintenance of cells. As far as we know, there is no report of the HA-MW into the disc and this report could help in future research about this GAG.

Our findings demonstrate that AFa has fewer viable cells/mm than AFp and older discs also have fewer cells which strengthens the data that in discs that are submitted to loading adaptive changes may result in disc degeneration[44,45], cell death begins at the fibrous annulus and apoptotic cells increase as stress and time increases[46]. Since about 80% of the compressive load passes through the vertebral bodies and the remaining 20% passes through the posterior elements[47], this load is expected to be different and higher in the anterior part of the vertebral body, which explains the smaller number of viable cells in this region and the great drop in GAG content in the AFa.

There is quite a large change in collagen organization between middle aged and elderly discs. A very large number of reddish fibres in all regions of middle-aged discs (Fig 4) was observed; the fibres in this group thus appear well organized and thick. In the older, more degenerate discs of old-aged group however, fibres tended to be greenish-yellowish, suggesting thinner and more disorganized fibrils[32]. Degradation of collagen[48] and other matrix components, leads to disorganisation of the matrix structure and loss of thicker organised collagen fibrils. We suggest that the changes we see result from this degradation.

There has been a focus on degenerative disc disease, where changes in the disc are thought to be the primary source of pain[49]. However, in other disorders such as fractures or spondylolisthesis, where minimally invasive surgery is used as the means of treatment, for these to succeed, it is absolutely necessary that the disc remains healthy. However, disc degeneration is also implicated in the development of spinal pathologies which affect the elderly in particular, such as spinal stenosis[50] and kyphoscoliosis[51]. With the increase in numbers of the elderly, and the growing incidence of spinal problems in this population, the results of this study show that more attention needs to be given to factors leading to degenerative changes in the disc, not only in middle age but right throughout life.

**Highlights**

- Cranial vertebral endplate is the main vascular route to the intervertebral disc
- Hyaluronic acid molecular weight is the same through the intervertebral disc after age 50’s
- Our results found that Di-6S/Di-4S increased with age and Di-6S was present in higher concentration only in the old-aged group
- there is a collagen disorganization in elderly discs predominating thinner fibres
- the number of viable cells/area was greater in the middle-aged discs
- AFa has fewer viable cells/mm and a greater drop in GAG content than AFp in both groups.

## Supporting information

S1 Fig Representative image of viable cells in the intervertebral disc. (*) presence of formazan crystals (metabolic active cells) around the blue nucleus stained with DAPI. (#) cell stained with DAPI without the formazan crystal–metabolic inactive cell. Images were acquired in the confocal microscope LSM780®. Bar = 10 μm.
(TIF)

S2 Fig Cell viability in different regions of the intervertebral disc in middle-aged and old-aged. Images acquired using confocal microscope LSM780®. Nucleus stained with DAPI (blue). (AFa) annulus fibrosus anterior; (NP) nucleus pulposus; (AFp) annulus fibrosus posterior. Bar = 30 μm.
(TIF)

S3 Fig Electrophoresis in PDA gels of pools of different intervertebral disc regions after enzymatic degradation. (A) Middle-aged. (B) Old-aged. (CS) chondroitin sulfate; (DS) dermatan sulfate; (HS) heparan sulfate; (Or) origin; (P) pattern; (H2O) water; (AC) chondroitinase AC; (ABC) chondroitinase ABC; (1) pool of anterior annulus fibrosus; (2) pool of nucleus pulposus; (3) pool of posterior annulus fibrosus.
(TIF)

S4 Fig Chromatogram of monodisperse hyaluronic acid standards. Chromatogram of monodisperse hyaluronic acid standards of known molecular weights: 2500kDa, 601kDa, 250 kDa, 150kDa and 100kDa analysed in Akta apparatus Purifier® OHpak SB- 805HQ (Shodex®) in series with column OHpak SB- 804HQ (Shodex®) 300 x 8.0 mm and detection UV at 205 nm after peak collected every 0.2 ml. Observe that the hyaluronic acid of higher molecular weight is eluted first than hyaluronic acid of different weights that are dislocated to the right as its weight reduces.
(TIF)

S5 Fig Chromatogram of the molecular weight analysis of the hyaluronic acid. Analysis using the Akta apparatus Purifier® OHpak SB- 805HQ (Shodex®) in series with column OHpak SB- 804HQ (Shodex®) 300 x 8.0 mm and detection UV at 205 nm after peak collected every 0.2 ml. (AFa1) Anterior Annulus Fibrosus Middle Aged; (NP1) Nucleus Pulposus Middle-Aged; (AFp1) Posterior Annulus Fibrosus Middle-Aged; (AFa2) Anterior Annulus Fibrosus Old-aged; (NP2) Nucleus Pulposus old-aged; (AFp2) Posterior Annulus Fibrosus old-aged.
(TIF)
