# Supplementary figures and images for: Changes in human intervertebral disc biochemical composition and bony end plates between middle and old age
Source: PLoS One. 2018 Sep 18;13(9):e0203932. doi: 10.1371/journal.pone.0203932 (PMC6144914; doi:10.1371/journal.pone.0203932)

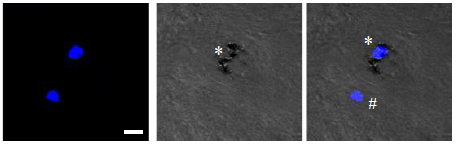

Supplement: S1 Fig — (*) presence of formazan crystals (metabolic active cells) around the blue nucleus stained with DAPI. (#) cell stained with DAPI without the formazan crystal–metabolic inactive cell. Images were acquired in the confocal microscope LSM780®. Bar = 10 μm. (TIF) [file pone.0203932.s001.tif]

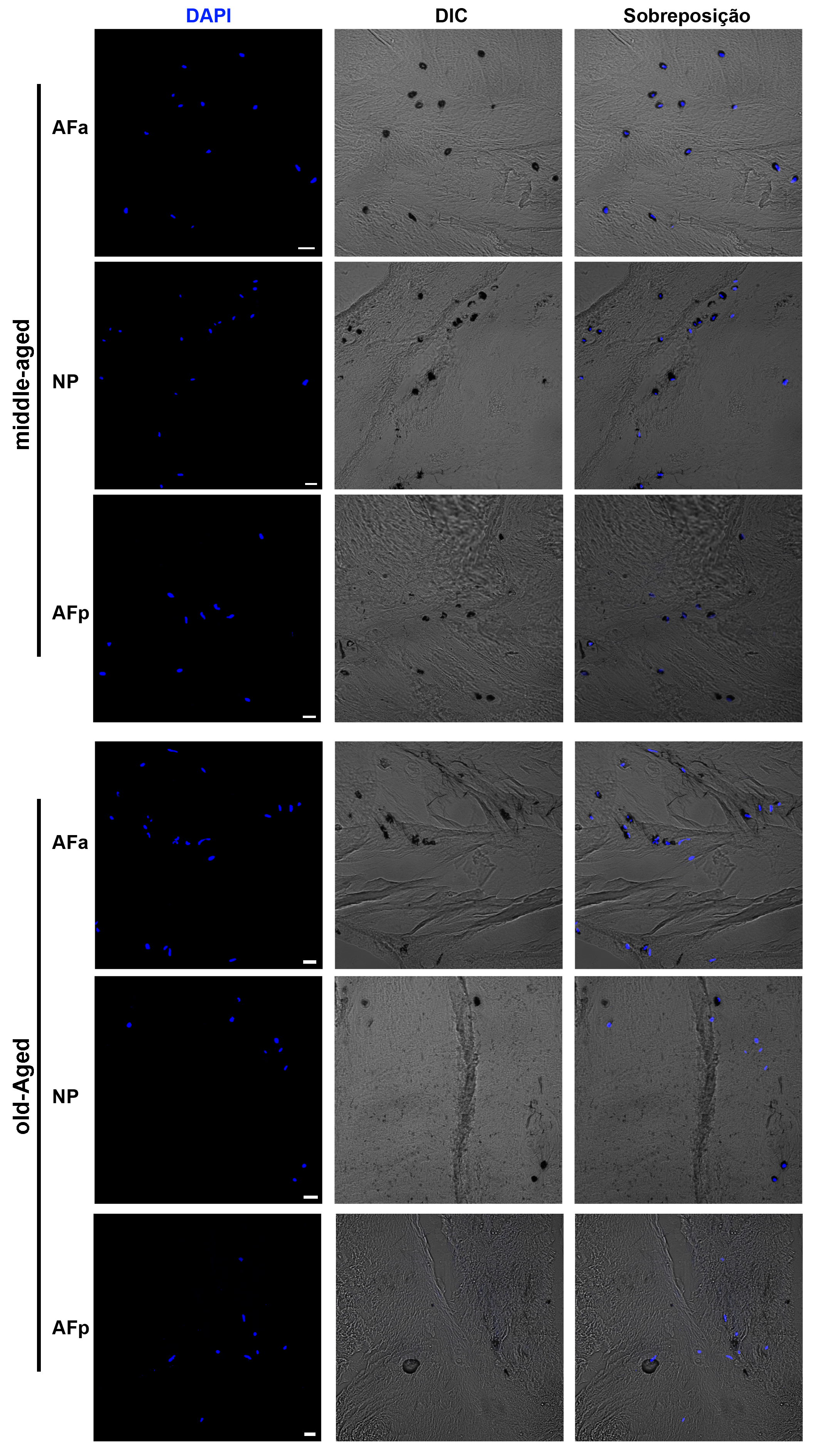

Supplement: S2 Fig — Images acquired using confocal microscope LSM780®. Nucleus stained with DAPI (blue). (AFa) annulus fibrosus anterior; (NP) nucleus pulposus; (AFp) annulus fibrosus posterior. Bar = 30 μm. (TIF) [file pone.0203932.s002.tif]

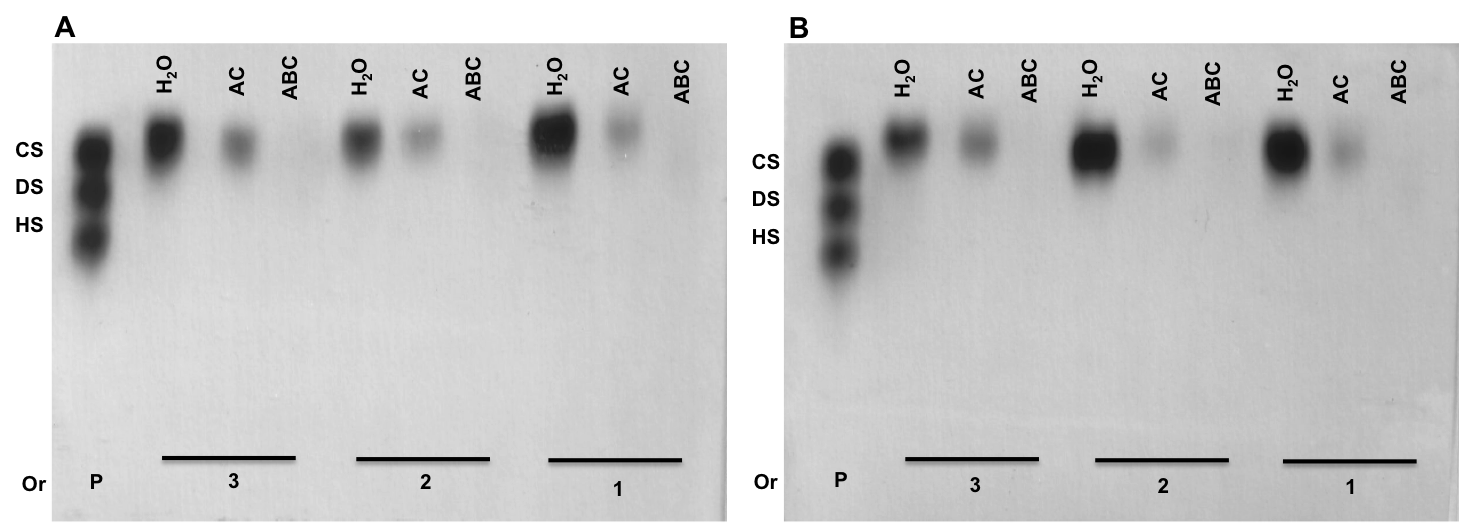

Supplement: S3 Fig — (A) Middle-aged. (B) Old-aged. (CS) chondroitin sulfate; (DS) dermatan sulfate; (HS) heparan sulfate; (Or) origin; (P) pattern; (H2O) water; (AC) chondroitinase AC; (ABC) chondroitinase ABC; (1) pool of anterior annulus fibrosus; (2) pool of nucleus pulposus; (3) pool of posterior annulus fibrosus. (TIF) [file pone.0203932.s003.tif]

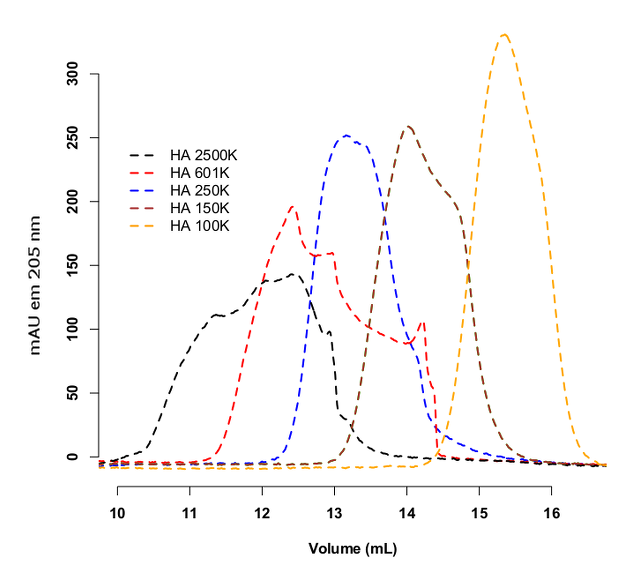

Supplement: S4 Fig — Chromatogram of monodisperse hyaluronic acid standards of known molecular weights: 2500kDa, 601kDa, 250 kDa, 150kDa and 100kDa analysed in Akta apparatus Purifier® OHpak SB- 805HQ (Shodex®) in series with column OHpak SB- 804HQ (Shodex®) 300 x 8.0 mm and detection UV at 205 nm after peak collected every 0.2 ml. Observe that the hyaluronic acid of higher molecular weight is eluted first than hyaluronic acid of different weights that are dislocated to the right as its weight reduces. (TIF) [file pone.0203932.s004.tif]

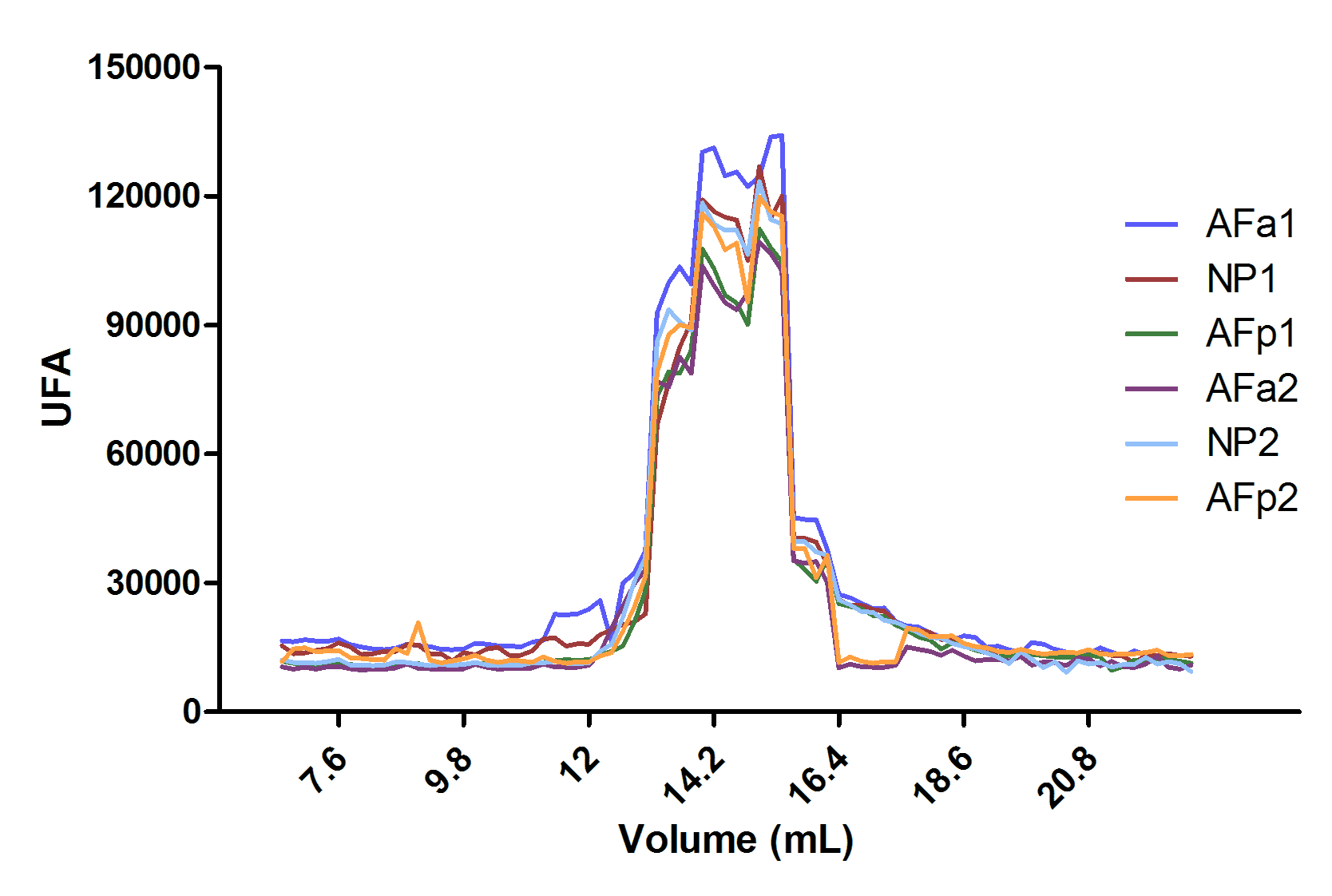

Supplement: S5 Fig — Analysis using the Akta apparatus Purifier® OHpak SB- 805HQ (Shodex®) in series with column OHpak SB- 804HQ (Shodex®) 300 x 8.0 mm and detection UV at 205 nm after peak collected every 0.2 ml. (AFa1) Anterior Annulus Fibrosus Middle Aged; (NP1) Nucleus Pulposus Middle-Aged; (AFp1) Posterior Annulus Fibrosus Middle-Aged; (AFa2) Anterior Annulus Fibrosus Old-aged; (NP2) Nucleus Pulposus old-aged; (AFp2) Posterior Annulus Fibrosus old-aged. (TIF) [file pone.0203932.s005.tif]
